# Interactional organization of WeChat-based pediatric consultations in China: a mixed-methods study of caregiver-physician exchanges

**DOI:** 10.3389/fpubh.2026.1942110

**Published:** 2026-09-09

**Authors:** Jijia Kuang, Xue Sun

**Affiliations:** 1 School of Foreign Languages, Chengdu University, Chengdu, China; 2 School of Humanities and Management Science, Southwest Medical University, Luzhou, China

**Keywords:** caregiver–physician communication, medical interaction, pediatric communication, sequence organization, WeChat group consultation

## Abstract

**Background:**

WeChat group consultations extend pediatric advice into everyday messaging, but their interactional organization is not well described.

**Objective:**

To identify recurring interactional stages, and clarification practices in asynchronous caregiver-physician exchanges.

**Methods:**

We analyzed 5,938 messages nested within 347 manually identified complete consultation episodes from one private pediatric WeChat group in Chengdu, China, collected from January to December 2024. A qualitatively led mixed-methods design combined manual sequence segmentation, descriptive analysis of message distribution and length, and targeted qualitative analysis of clarification sequences.

**Results:**

Four recurrent stages were identified: problem initiation, clinical data collection, diagnostic advice, and closure. Opening and illness presentation were combined in 69.45% of episodes (241/347), and diagnosis with treatment recommendation in 85.88% (298/347). Caregivers produced more messages overall than physicians (3,273 vs. 2,665), with substantially longer and more variable messages during problem initiation (median = 9 vs. 3; M = 17.91 vs. 3.50; IQR = 14.75 vs. 3) and clinical data collection (median = 10 vs. 7; M = 14.22 vs. 9.29; IQR = 11 vs. 7). Message-length distributions became more comparable during diagnostic advice (median = 10 for both; IQR = 12 vs. 13). Clarification sequences were identified in both directions: 293 physician-led sequences during clinical data collection and 686 caregiver-initiated sequences during diagnostic advice, progressively transforming underspecified symptom reports into more specified clinical accounts.

**Conclusion:**

The findings demonstrate how asynchronous messaging redistributes communicative activities between caregivers and physicians, with clarification as a recurrent mechanism for specifying clinically relevant information. Structured symptom prompts, clearer message threading, and explicit escalation guidance warrant testing in future intervention research. This study provides an empirical basis for understanding the sequential organization of asynchronous pediatric consultations and offers actionable directions for communication design in digital health services.

## 1 Introduction

The rapid integration of digital technologies into healthcare has transformed the delivery of medical services, improving accessibility, flexibility, and continuity of communication (1–3). These changes have also reshaped how healthcare providers and patients exchange information, extending clinical communication beyond conventional face-to-face encounters (4). Among these innovations, WeChat-based medical consultation groups in China represent a distinctive form of digitally mediated healthcare communication, embedding clinical interactions within an everyday messaging environment (5–7). This model allows caregivers and healthcare providers to exchange health information asynchronously through a familiar and accessible communication platform. Compared with face-to-face encounters, digitally mediated communication requires clinical information to be coordinated through text-based messages and platform-mediated interaction (8, 9). In China, WeChat has become an established channel for physician–patient communication, including group-based interactions (10). In such asynchronous settings, clinical information is exchanged across multiple text-based messages rather than through a single, continuously unfolding encounter. This may alter how information is introduced, clarified, and taken up across the course of a consultation. Examining the interactional organization can provide insight into how pediatric consultations are accomplished in digitally mediated, group-based healthcare settings, and is therefore important for understanding how caregivers’ health concerns are developed into clinically actionable exchanges in digitally mediated pediatric care.

## 2 Literature review: the architecture of institutional talk in digital space

The study of medical communication has long been informed by the theoretical framework of institutional discourse analysis, which examines how interaction is shaped by, and in turn constitutes, institutional contexts. Unlike ordinary conversation, institutional discourse is oriented toward institutionally defined goals, constrained by participant roles and communicative norms, and organized through context-specific interactional practices. The seminal work of Drew and Heritage established that institutional encounters are characterized by goal orientation, constraints on participants’ contributions, and distinctive inferential frameworks, demonstrating how institutional tasks are accomplished through structured communicative practices (11).

Within the medical setting, a quintessential institutional domain, this framework has provided important insights into the organization of medical encounters. Research on medical communication has demonstrated that clinical consultations follow recognizable interactional structurers, including opening sequences, history-taking, examination, diagnosis, and treatment recommendation and closure (12). Earlier clinical communication models similarly emphasized the phased organization of physician–patient encounters and the importance of interactional efficiency in accomplishing diagnostic and therapeutic tasks (13). This structured interaction is also a key site for the enactment of inherent power asymmetries between physicians and patients, where clinicians exercise authority through questioning, topic control, and diagnostic pronouncements, while patients often occupy the role of information providers and advice recipients (14, 15). Medical consultation therefore represents a specific activity type in which participants operate within shared expectations regarding professional expertise, patient roles, and institutional objectives (16). Consequently, medical communication is not merely a neutral exchange of information but a process through which professional identities, epistemic authority, and healthcare relationships are continuously constructed and negotiated.

Beyond its institutional organization, health communication scholarship has emphasized that clinical interaction performs multiple functions beyond information transmission. Patient–provider communication serves instrumental purposes, such as diagnosis and treatment decision-making, while also performing relational and emotional functions that influence patient participation and healthcare experiences. From an ecological perspective, medical communication simultaneously involves information exchange, relationship development, emotional management, and decision-making across multiple levels of healthcare interaction (17). Similarly, research on physician–patient communication has demonstrated that task-oriented and socio-emotional dimensions coexist within medical encounters, with physicians’ communication styles, information provision, and relational practices influencing patient participation, satisfaction, and perceptions of care (18). However, much of this influential research has been grounded in synchronous, co-present, and predominantly dyadic face-to-face encounters. Recent research comparing face-to-face and teleconsultations suggests that, although clinical communication may retain broadly comparable functions across modalities, the ways in which interactional practices are realized can vary according to the communication medium (19). These differences make it necessary to reconsider how established models of medical communication apply to asynchronous and digitally mediated healthcare environments.

Against this backdrop, the rise of internet-based communication subsequently brought Computer-Mediated Communication (CMC) and health communication into closer contact, shifting scholarly attention toward online health communities and digitally mediated support environments. Research on virtual communities has identified electronic support groups as important spaces for peer interaction, knowledge sharing, and social support (20), while subsequent studies have suggested that participation in such communities can facilitate empowerment by enabling individuals to exchange experiences, access collective knowledge, and develop supportive relationships (21).

CMC research has also highlighted how text-based mediation alters the conditions under which interpersonal communication takes place. Hyperpersonal Communication Theory, for example, suggests that the reduced availability of nonverbal cues in mediated interaction can encourage selective message construction and impression management (8). In healthcare contexts, such mechanisms may affect how professional identities are presented and how patients formulate and interpret health-related information. Nevertheless, the implications of CMC for healthcare communication extend beyond interpersonal impression formation, particularly when communication is embedded within institutional relationships and unfolds asynchronously.

Asynchronous communication creates distinctive interactional conditions. Delayed responses may provide participants with additional time to organize information and formulate responses, but they may also produce uncertainty when immediate clarification or reassurance is expected (22). As digital platforms have become increasingly embedded in healthcare practices, scholars have consequently emphasized that communication technologies should not be treated as neutral channels. Platform architectures and affordances can shape participation, message visibility, persistence, accessibility, and interactional practices (23). In healthcare communication, features such as persistent message histories, group structures, @mentions, multimedia functions, notifications, and asynchronous response patterns may influence how information is produced, organized, retrieved, and interpreted. These features are particularly consequential for medical interactions because they may alter the sequential organization through which participants establish symptoms, clarify information, formulate clinical assessments, and deliver recommendations.

WeChat represents a particularly important case of digitally mediated healthcare communication because it integrates instant messaging, social networking, and service functions within a single platform. Unlike conventional face-to-face consultations or anonymous online health forums, WeChat healthcare groups can combine institutional medical authority with everyday messaging practices. Within this environment, caregivers and physicians exchange health information through multiple text-based contributions. Research on mobile and instant messaging communication has shown that messaging platforms can blur conventional boundaries between professional and everyday communication, creating both opportunities and challenges for professional communication, privacy management, and expectations of accessibility (24). Studies examining instant messaging applications such as WhatsApp and WeChat have reported potential benefits including improved accessibility, continuity of communication, patient support, and opportunities for patient-generated information sharing (25). At the same time, researchers have identified challenges related to information overload, privacy protection, professional boundary management, and unequal access to digital healthcare resources (26, 27).

Although this body of research demonstrates the functional value and practical challenges of messaging-based healthcare communication, much of it remains focused on user experiences, thematic content, adoption, or service-level outcomes. Comparatively less attention has been paid to the micro-level interactional processes through which asynchronous medical consultations are organized. In particular, limited research has examined how caregivers formulate symptom descriptions, how physicians seek clarification or respond to information provided by caregivers, and how sequential interaction facilitates clarification in group-based pediatric consultations.

The existing literature therefore reveals a gap between established approaches to clinical interaction and emerging forms of digitally mediated healthcare communication. Institutional discourse analysis provides sophisticated tools for examining the sequential organization of medical encounters, role relations, and participant roles and the sequential organization of medical encounters, but these approaches were largely developed around synchronous and co-present interaction. Digital health research, by contrast, has increasingly documented the accessibility, continuity, and practical value of messaging platforms, yet has comparatively less frequently examined how participants accomplish the interactional work of medical consultation through short, asynchronous, and multiple messages. This gap is particularly important in WeChat-based pediatric consultations, where consultation episodes unfold through sequences of asynchronous messages and where individual consultation episodes must remain intelligible across sequences of asynchronous messages. The central empirical problem is therefore not simply whether digital communication improves healthcare accessibility, but how participants organize and accomplish institutional medical activities when consultation unfolds through asynchronous messages rather than face-to-face interaction. Specifically, caregivers may provide symptoms and contextual information across multiple short messages, while physicians may ask follow-up questions, provide clinical assessments, and offer recommendations across successive exchanges. Examining these processes can reveal how communicative activities and participant roles are organized across the consultation.

Research on clinical interaction, teleconsultation, and computer-mediated communication provides useful conceptual resources for understanding these transformations, while studies of WeChat and WhatsApp have documented the broader functional and organizational implications of instant messaging in healthcare (8, 11, 12, 17, 18, 20, 21, 25, 28, 29). However, these research traditions have not yet sufficiently explained how a shared asynchronous group chat maintains the coherence of individual pediatric consultations when short messages, delayed responses, and concurrent interaction threads redistribute informational work between caregivers and physicians.

To address this gap, the present study examines WeChat-based pediatric consultations as digitally mediated institutional interactions drawing on insights from institutional discourse analysis and health communication research. By systematically identifying recurring interactional structures and communicative practices within WeChat-based pediatric consultations, this study extends existing theories of medical interaction into digitally mediated environments and provides empirical insights into how instant messaging platforms reshape caregiver–physician communication. Specifically, the study investigates the following research questions: *RQ1*: What recurring interactional activities characterize complete caregiver–physician consultation episodes? *RQ2*: How are communicative activities and message contributions distributed across consultation stages and participant roles? *RQ3*: How are clarification sequences organized across consultation stages and participant roles?

## 3 Materials and methods

### 3.1 Corpus description

The corpus consisted of chat records from January to December 2024 from a pediatric WeChat consultation group established by a children’s hospital in Chengdu, a major city in western China. The group operated as a hospital-based online pediatric consultation service designed to extend medical communication beyond conventional face-to-face encounters. Caregivers could post pediatric health-related questions and provide relevant information, including symptom descriptions, medical histories, examination results, and other contextual details. Physicians on duty responded asynchronously according to their clinical schedules and availability. Each consultation was subject to a fixed service fee of RMB 20, indicating that the interaction constituted a formally organized healthcare service rather than informal health-related discussion.

### 3.2 Data cleaning and consultation episode reconstruction

#### 3.2.1 Message-level data cleaning

The original WeChat archive contained 8,159 records. Each record represented one individual message entry generated within the group chat, including text messages, multimedia-related entries, and system-generated records captured during data export. Thus, a record was treated as a message-level entry rather than as a complete conversational turn, consultation episode, or participant unit.

Data processing proceeded in two stages before episode-level annotation, ensuring that the final corpus consisted only of communicatively interpretable messages relevant to caregiver–physician consultation.

In the first stage, technical cleaning was conducted to remove records that did not constitute independent communicative messages. This included blank entries, duplicated system-generated records, formatting inconsistencies, incomplete export records, and other technical artifacts introduced during data extraction. A total of 1,596 records were removed at this stage, leaving 6,563 records in the preliminary communication dataset. Following technical cleaning, the remaining 6,563 records were screened for their relevance to caregiver–physician pediatric consultation. Messages were excluded when they were unrelated to the consultation process and did not contain a caregiver’s description of a child’s symptoms, health condition, or a request for clinical advice. A total of 625 records were excluded. These consisted of 78 hospital-generated records, including physician duty/availability announcements (*n* = 54) and hospital promotional announcements (*n* = 24), and 547 participant-generated non-consultation records. The latter included questions concerning medication prescribed during offline hospital visits (*n* = 194), requests to add friends to the consultation group (*n* = 8), questions about how to initiate a consultation (*n* = 22), inquiries about offline laboratory testing or report-interpretation schedules (*n* = 48), payment-related administrative exchanges (*n* = 11), unrelated advertisements (*n* = 18), requests to review reports from other hospitals (*n* = 64), and requests to purchase or substitute medication (*n* = 182). After content-based screening, 5,938 consultation-related messages remained for analysis.

#### 3.2.2 Operational definition of a consultation episode

Because the consultation service operated through a shared group chat, messages from different caregivers could occur concurrently. Consequently, chronological adjacency alone could not be assumed to indicate participation in the same consultation. A consultation episode was therefore defined as a coherent caregiver–physician interaction concerning one consultation request, rather than simply as a temporally contiguous sequence of messages.

An episode began when a caregiver initiated a pediatric health-related consultation by describing the child’s symptoms, health concerns, or related clinical information. It continued through the subsequent caregiver–physician exchanges concerning the same consultation and ended when the interaction reached an identifiable interactional closure. The service-specific payment procedure provided an important contextual marker of closure. Caregivers were required to complete a fixed consultation payment after receiving the physician’s advice. Accordingly, explicit indicators such as requesting or providing the payment code, indicating that payment had been completed, or expressing thanks to the physician following the consultation were treated as evidence that the consultation interaction had reached closure. These indicators were interpreted as markers of interactional completion, rather than as evidence that the child’s medical condition had been clinically resolved. An episode was therefore considered complete when both its initiation and its interactional closure could be identified within the archived records. The term complete consultation episode refers specifically to the completeness of the observable interactional sequence in the corpus and does not imply diagnostic completeness, treatment completion, or resolution of the patient’s clinical condition.

#### 3.2.3 Resolution of concurrent and overlapping threads

To address the possibility that several consultations could occur simultaneously within the shared group, episode attribution was based on multiple interactional and contextual cues rather than on message timing alone.

Where available, reply or quotation links and @mentions were treated as direct evidence connecting a message to a preceding contribution. When explicit platform-level linking information was unavailable, temporal proximity was considered jointly with semantic continuity. Semantic continuity was assessed by examining whether successive messages referred to the same child’s symptoms, clinical concern, previously supplied information, question, or physician response. For example, a caregiver’s follow-up description of the same symptoms, a physician’s response addressing those symptoms, or a caregiver’s clarification of information requested by the physician was treated as evidence of continuity. Conversely, the occurrence of another caregiver’s unrelated symptom description or clinical request between two messages did not automatically divide an existing episode if the surrounding interactional evidence indicated that the messages belonged to the same consultation.

Importantly, temporal proximity alone was not considered sufficient to establish episode membership. No arbitrary fixed maximum time-gap threshold was imposed because the consultation service was asynchronous and participants could respond according to their availability. Temporal proximity was therefore treated as one contextual indicator within a broader episode-attribution procedure rather than as an independent boundary rule.

#### 3.2.4 Manual episode identification and participant verification

Following message-level screening and application of the episode-attribution criteria, two researchers with expertise in discourse analysis manually reviewed the consultation records and identified complete consultation episodes. The annotation focused on the sequential relationship among messages and the continuity of the clinical interaction rather than on the chronological grouping of all messages appearing in the group.

This procedure identified 347 complete consultation episodes containing the 5,938 analyzable messages. The episodes involved 141 unique caregivers and 10 physicians. Some caregivers contributed to more than one consultation episode during the study period; therefore, the number of consultation episodes does not correspond to the number of unique participants (Table 1).

**Table 1:** Data cleaning, screening, and corpus construction.

Stage	Description	Number	Percentage (%)
Raw WeChat archive entries	Original exported records	8,159	8.64
After technical cleaning	Removed blank lines, duplicates, system artifacts	6,563	3.84
After content screening	Removed advertisements, administrative notices, non-consultation messages	5,938	31.04
Content screening	Requests to add friends to the consultation group	8	1.28
	Questions about how to initiate a consultation (e.g., whether to consult by directly sending a message)	22	3.52
	Questions about schedules for offline laboratory testing or report interpretation	48	7.68
	Payment-related administrative exchanges (e.g., mistaken receipt and return of a patient’s payment/red packet)	11	1.76
	Unrelated advertisements, including messages subsequently warned against by physicians	18	2.88
	Requests to review or interpret laboratory/examination reports from other hospitals	64	10.24
	Requests to purchase or substitute medication	182	29.12
Content screening Total	Records unrelated to caregiver–physician pediatric consultation	625	100
Complete consultation episodes	Episodes meeting inclusion criteria	347	
Caregivers	Unique caregivers consulting in the group	141	
Physicians	Unique physicians	10	
Final analytic messages	Messages included in analysis	5,938	

Content screening was conducted after technical cleaning. Records were excluded when they were unrelated to pediatric consultation and did not contain a caregiver’s description of a child’s symptoms, health condition, or related request for clinical advice. The excluded records comprised hospital-generated announcements and participant-generated exchanges that did not constitute the caregiver–physician clinical consultation analyzed in this study.

### 3.3 Analytical framework and coding procedures

The study adopted a qualitatively led mixed-methods design, combining manual sequential analysis of complete consultation episodes with descriptive quantitative analysis of message distribution and length. The analytical framework was developed to address three questions: how complete pediatric consultations were sequentially organized, how interactional activity was distributed across stages and participant roles, and how clarification sequences contributed to the distribution of informational work.

The analysis proceeded in three interconnected steps. First, 347 complete consultation episodes were manually identified and initially examined using the six-stage consultation framework proposed by Byrne and Long: opening, illness statement, data collection, diagnosis, treatment recommendation, and conclusion. Second, the initial six-stage coding was examined for the extent to which theoretically distinct activities could be reliably distinguished in the asynchronous WeChat environment. Where activities were repeatedly co-realized and could not be reliably separated, they were consolidated into broader interactional stages, resulting in a four-stage framework. Third, the adapted four-stage framework was systematically coded across the full analytic corpus of 5,938 messages, after which descriptive measures of message production and message length were calculated., Selected clarification sequences were examined qualitatively to investigate the interactional mechanisms underlying repeated elicitation and advice clarification.

#### 3.3.1 Identification of interactional stages

The stage-identification procedure was informed by established models of medical consultation organization. In particular, the six-stage model proposed by Byrne and Long—opening, illness statement, data collection, diagnosis, treatment recommendation, and conclusion—provided an initial theoretical framework for examining the organization of pediatric consultations (13).

The six-stage framework was not imposed as the final coding scheme. During initial coding of the 347 complete consultation episodes, the research team and two trained coders examined whether each theoretically defined activity could be reliably identified as an independently observable interactional stage in the asynchronous WeChat data. Activities that were consistently co-realized or could not be reliably distinguished as separate stages were considered for consolidation. The resulting stage structure was therefore determined through iterative comparison between the theoretical framework and the interactional organization observed in the corpus.

Four broader stages were subsequently defined: (1) Problem initiation, in which the caregiver introduces the child’s health concern, symptoms, or reason for consultation; (2) Clinical data collection, in which caregivers and physicians exchange additional clinical information, including responses to questions and clarification of symptoms or relevant contextual information; (3) Diagnostic advice, in which physicians provide clinical assessments, diagnostic interpretations, treatment recommendations, medication advice, or management guidance; and (4) Closure, in which the consultation moves toward completion through acknowledgement, confirmation, thanks, payment-related completion, or other recognizable closing activities.

Stage assignment was based on the dominant interactional activity and its relationship to surrounding messages rather than solely on message position. This procedure was particularly important in the asynchronous group-chat setting, where individual messages could contain more than one communicative action. At the episode level, the four stages were identified as the recurrent organizational units of complete consultations; at the message level, each message was assigned to the stage that best represented its dominant interactional activity within the surrounding sequence.

#### 3.3.2 Sequence-stage coding and inter-coder reliability

Following an initial exploratory analysis of the corpus, a four-stage sequence framework was developed and formalized in a predefined coding manual. The complete analytic corpus was subsequently coded at the message level. The individual WeChat message was used as the basic coding unit.

Each message was assigned to the interactional stage that best represented the activity being accomplished within the surrounding sequence. The coding scheme comprised four stages: (1) problem initiation, (2) clinical data collection, (3) diagnostic advice, and (4) closure. Stage assignment took into account the preceding and subsequent messages, the communicative activity being performed, the developing topic of the consultation, and the participant’s role in the interaction. Messages were therefore not classified solely on the basis of isolated linguistic content or speaker identity.

Particular attention was given to stage boundaries. A new stage was recognized when the dominant interactional activity shifted. For example, a sequence of symptom description and clinical elicitation was considered to transition into diagnostic advice when the physician moved from gathering or clarifying information to clinical interpretation, diagnostic assessment, or treatment recommendation. Closure was identified when the interaction shifted from clinical activity to signals of consultation completion, such as expressions of thanks, indications that no further questions remained, or other explicit completion signals. Where a message contained activities potentially associated with two adjacent stages, the message was assigned according to the dominant interactional activity accomplished within the surrounding exchange. This priority rule was applied consistently across the corpus.

Participant role provided an important contextual cue for stage identification but was not treated as a deterministic coding rule. Caregivers most commonly initiated consultations by presenting the child’s health concern, whereas physicians typically initiated clinical data collection through follow-up questions and initiated diagnostic advice through clinical assessment or recommendations. Caregivers frequently participated in closure by indicating appreciation, confirming that no further questions remained, or otherwise signaling completion. These recurrent associations between participant roles and consultation stages were treated as interactional patterns rather than categorical coding rules.

The stage coding was conducted independently by two trained researchers with expertise in discourse analysis. A predefined coding manual specified the operational definition of each stage, inclusion and exclusion criteria, stage-boundary rules, and procedures for ambiguous cases. The two coders independently coded all 5,938 analyzable messages. They agreed on 5,932 messages and disagreed on 6, resulting in a raw agreement of 99.90%. Cohen’s κ was calculated separately to account for chance agreement and was κ = 0.998, indicating near-perfect inter-coder agreement.

The six discrepant cases were reviewed with reference to the surrounding interactional context and the coding manual. All six disagreements concerned the boundary between clinical data collection and diagnostic advice. Cases that remained difficult to resolve were discussed and adjudicated by a third researcher with expertise in medical discourse analysis. The final analytic dataset reflects the adjudicated coding.

The episode-level and message-level analyses were therefore treated as complementary rather than interchangeable. The episode-level analysis established the presence of the four broader interactional stages across the 347 consultation episodes, whereas the message-level coding quantified how caregiver and physician messages were distributed across those stages. The message counts reported subsequently should therefore be interpreted as descriptive distributions within the four-stage organization, rather than as evidence that each individual episode followed four rigidly bounded phases.

#### 3.3.3 Targeted qualitative sequence analysis

Clarification sequences were operationalized as interactional sequences in which one participant’s preceding contribution generated a need for additional specification, explanation, or elaboration, followed by a subsequent contribution that responded to the clarification request. Two forms were distinguished according to interactional direction and consultation stage. Physician-led clarification was identified during Clinical Data Collection when a physician asked a follow-up question concerning symptoms, duration, frequency, associated symptoms, previous treatment, feeding, medication use, or other clinically relevant information, and the caregiver subsequently provided, specified, corrected, or elaborated the requested information. Caregiver-initiated clarification was identified during Diagnostic Advice when a caregiver requested further explanation, interpretation, or practical guidance following a physician’s diagnostic assessment, treatment recommendation, medication instruction, or management advice, and the physician subsequently responded to the request.

The coding unit was the clarification-bearing message post, whereas the clarification sequence was the unit used to identify and analyze the interactional pattern. Accordingly, message-post frequencies and sequence frequencies were reported separately. The focused qualitative sequence analysis was conducted to address RQ3. It did not attempt to assign a comprehensive pragmatic-function label to every message; rather, it examined the two predefined clarification patterns that were directly relevant to the research question.

For physician-led clarification, the analysis examined sequences in which a physician requested additional or more specific clinical information following an incomplete, vague, or underspecified caregiver report. Particular attention was given to sequences involving repeated physician questions and subsequent caregiver responses. The analysis examined how successive questions elicited information concerning symptom occurrence, duration, frequency, associated symptoms, feeding, medication use, and other clinically relevant details. The purpose was to characterize how physicians progressively elicited more specific clinical information from caregivers’ initial descriptions.

For caregiver-initiated clarification, the analysis examined sequences in which a caregiver requested additional explanation or specification after receiving a physician’s diagnostic assessment, treatment recommendation, medication instruction, or management advice. Particular attention was given to questions concerning the meaning of a diagnosis, how medication or treatment should be used, whether existing medication should be continued, and what subsequent action should be taken. The analysis therefore focused on how caregivers sought clarification of diagnostic and management information and how physicians responded to these requests.

### 3.4 Quantitative analysis

Quantitative analysis was descriptive and focused on message production and message length across the four interactional stages and the two participant roles.

#### 3.4.1 Message distribution

The number of messages produced by caregivers and physicians was calculated for the complete corpus and separately for each interactional stage. These counts were used to describe how communicative activity was distributed between the two participant groups and how this distribution changed across the consultation.

Because the corpus consisted of a shared group-chat environment, message counts were calculated at the level of individual messages rather than treating participants or consultation episodes as equivalent units. Participant counts and episode counts were reported separately to maintain the distinction between unique participants, consultation episodes, and individual messages.

#### 3.4.2 Message length

Message length was measured by the number of Chinese characters contained in each message. Because the original WeChat data consisted primarily of Chinese-language messages, character counts were calculated directly from the original Chinese text.

Character length was calculated in Microsoft Excel using the following formula: =LENB(cell)-LEN(cell)`.

This procedure was used to estimate the number of Chinese characters in each message by exploiting the difference between byte length and character length in the Chinese-language dataset. The calculation was performed on the original Chinese messages rather than on English translations or glosses.

Message-length distributions were then summarized separately by participant role and consultation stage. Because message length was not normally distributed and showed substantial variation across messages, the analysis emphasized medians and interquartile ranges (IQRs). Means were also reported where useful to describe overall differences in message length.

The resulting descriptive statistics were used to examine whether caregivers and physicians differed in their message production patterns and whether these differences varied across the four interactional stages.

## 4 Results

### 4.1 The recurrent four-stage structure of WeChat-based pediatric consultations

The analysis revealed a recurrent four-stage interactional structure underlying WeChat-based pediatric consultations: problem initiation, clinical data collection, diagnostic advice, and closure. The consolidation of the original six consultation activities was supported by recurrent patterns of co-realization observed in the corpus (Table 2).

**Table 2:** Empirical consolidation of the initial six-stage consultation framework into a four-stage interactional framework.

Initial six-stage framework	Adapted four-stage framework	Empirical basis for consolidation
Opening + Illness statement	Problem initiation	Co-realized or interactionally inseparable in 241/347 episodes (69.45%)
Data collection	Clinical data collection	Retained as a distinguishable interactional activity
Diagnosis + Treatment recommendation	Diagnostic advice	Co-realized in 298/347 episodes (85.88%); 53 episodes contained treatment recommendations without a separately identifiable diagnostic statement
Conclusion	Closure	Retained as a distinguishable interactional activity

The first pattern concerned the relationship between opening and illness statement. In 241 of the 347 episodes (69.45%), these activities were co-realized rather than occurring as separately identifiable stages. Caregivers frequently established contact with the physician while immediately presenting the child’s symptoms or health concern. For example:

*Extract 1*. Integrated problem initiation.

*Caregiver*: @医生 您好, 我家宝宝昨天晚上开始发烧, 今天还有点咳嗽, 最高38.7 °C. @Doctor, hello. My baby started having a fever last night and still has a bit of a cough today. The highest temperature was 38.7 °C.

As illustrated in Extract 1, the caregiver simultaneously establishes contact with the physician and introduces the child’s presenting complaint. The greeting, symptom onset, principal symptoms, and an indication of severity are contained within the same contribution, making the two activities difficult to distinguish as separate interactional stages. This pattern was recurrent across the corpus, supporting their treatment as a broader problem initiation stage.

A second pattern concerned the relationship between diagnosis and treatment recommendation. These activities were co-realized in 298 of the 347 episodes (85.88%), with physicians frequently combining clinical assessment and management advice within the same response sequence. In a further 53 episodes (15.27%), treatment recommendations were provided without a separately identifiable diagnostic statement. This pattern indicates that diagnostic assessment did not consistently constitute an independently observable stage preceding treatment recommendation.

*Extract 2*. Integrated diagnostic advice.

*Physician*: 目前看考虑病毒感染, 精神状态好的话可以先观察, 多喝水, 如果持续高烧不退建议到医院进一步检查. Based on the current presentation, it is likely to be a viral infection. If the child is in good spirits, you can observe the child at home for now and encourage plenty of fluids. If the fever remains high and does not subside, further evaluation at a hospital is recommended.

In Extract 2, the physician first provides a clinical assessment (“考虑病毒感染” It is likely to be a viral infection) and then immediately specifies corresponding management actions, including observation, hydration, and further medical evaluation if the fever persists. The diagnostic and treatment-oriented functions are therefore accomplished within the same response rather than through two distinct interactional stages. This pattern was sufficiently recurrent to warrant their consolidation into a single category of diagnostic advice. Importantly, this category does not imply that diagnosis and treatment recommendation are conceptually identical. Rather, it captures their frequent interactional co-realization in the WeChat corpus.

Taken together, the findings indicate that problem initiation, clinical data collection, diagnostic advice, and closure. Represent the recurrent interactional organization of the consultation episodes. These stages are best understood as broader interactional units rather than rigid message-level categories. Table 3 provides an illustrative example of how the four stages unfold across a complete consultation episode.

**Table 3:** An illustrative four-stage consultation.

Person	Chat posts	Sequences
咨询人1: Caregiver 1	@医生, 刚午睡烧高了, 三十八度三, 手脚还凉应该还会高@Doctor 1, (my child) just took a nap and his fever went up. It’s 38.3 Degrees Celsius. His hands and feet are very cold. Maybe the fever will rise further.	Problem initiation
咨询人1: Caregiver 1	鼻子一上午喷的没那么塞的His nose wasn’t that stuffy this morning for being sprayed (medicine).	Problem initiation
咨询人1: Caregiver 1	下午醒就这样咳嗽, 精神暂时还行He started coughing like this after his noon nap. Still having good energy for now.	Problem initiation
医生1: Doctor 1	咳嗽很频繁吗? How frequent is the coughing?	Clinical data collection
咨询人1: Caregiver 1	他一吸鼻子就会这样咳几声 He coughs a few times like that whenever he sniffs.”	Clinical data collection
咨询人1: Caregiver 1	不频繁, 就是一吸鼻子会咳几声, 现在就是起烧了了It’s not that frequent—just a few coughs when inhaling through the nose. But now the fever is starting.	Clinical data collection
咨询人1: Caregiver 1	嗓子下午我又给看了看, 可能烧的原因, 比上午略红点I checked his throat again this afternoon—possibly due to the fever, it looks a bit redder than in the morning.	Clinical data collection
医生1: Doctor 1	好的, 可以买个氨酚麻美干混悬剂口服Ok, you can buy an acetaminophen/pseudoephedrine/dextromethorphan dry suspension for oral use.	Diagnostic advice
医生1: Doctor 1	一般是咽喉部炎症引起的发烧和咳嗽This kind of fever and cough is usually caused by throat inflammation.	Diagnostic advice
咨询人1: Caregiver 1	那继续您昨早的药还是换So, should I continue with last morning’s medication from your hospital or switch?	Diagnostic advice
医生1: Doctor 1	如果咳嗽非常频繁的话, 可以去买美敏伪麻溶液或者氨酚麻美干混悬剂, 如果都买不到就加个右美沙芬口服液. If the cough is very frequent, you can buy a chlorpheniramine/pseudoephedrine/dextromethorphan oral solution or an acetaminophen/pseudoephedrine/dextromethorphan dry suspension. If neither is available, you can add a dextromethorphan oral solution.	Diagnostic advice
医生1: Doctor 1	对了备一瓶退烧药哈, 如果发烧超过38.5口服. Also, keep a bottle of fever reducer on hand—if the fever goes above 38.5 °C, take it orally.	Diagnostic advice
咨询人1: Caregiver 1	退烧药家里有We already have fever reducer at home.	Diagnostic advice
咨询人1: Caregiver 1	谢谢医生, 发码Thank you, doctor. Please send the payment code.	Closure
医生1: Doctor 1	不客气You are welcome	Closure

Table 3 illustrates how the four stages unfold within a complete consultation episode. The episode begins with the caregiver’s presentation of the child’s immediate health concerns, followed by exchanges through which additional clinical information is elicited and provided. The interaction then moves into diagnostic advice, where the physician provides clinical assessment and management recommendations, before progressing to closure. Although individual episodes varied in length and message organization, this overall progression was recurrent across the corpus.

The stages also differed in the distribution of caregiver and physician contributions. Caregivers contributed more extensively during problem initiation and clinical data collection, where they introduced symptoms and provided additional information in response to physicians’ questions. Diagnostic advice, in contrast, was primarily physician-led and involved clinical assessment, medication advice, and management recommendations. These differences indicate that the four-stage structure was associated with different patterns of communicative participation across consultation stages.

### 4.2 Stage-specific distribution of messages and interactional contribution

Having established the recurrent four-stage structure of WeChat-based pediatric consultations, the next analysis examined how messages were distributed across these stages and how caregivers and physicians differed in their interactional contributions. The quantitative analysis indicates that the four stages were not equally represented in the corpus. More importantly, the distribution of message volume and message length varied systematically according to both interactional stage and participant role as shown in Table 4. These quantitative patterns are consistent with the interactional organization identified through sequence analysis, in which information-providing activities are more prominent in caregiver contributions and diagnostic and management activities are more strongly associated with physician contributions.

**Table 4:** Message distribution and length by consultation stage and participant.

Panel A. Message-level statistics by consultation stage						
Participant	Statistic	Problem initiation	Clinical data collection	Diagnostic advice	Closure	Total
Caregiver	Median	9	10	10	4	
	Mean	17.9	14.22	13.06	6.06	
	Q1	6	6	5	2	
	Q3	20.75	17	17	7	
	Interquartile range	14.75	11	12	5	
	Posts number	312	1,159	1,491	311	3,273 (in total)
Physician	Median	3	7	10	7	
	Mean	3.50	9.29	15.71	7.89	
	Q1	2	4	5	4	
	Q3	5	11	18	10	
	Interquartile range	3	7	13	6	
	Posts number	153	788	1,413	311	2,665 (in total)

Panel B. Participant-level summary statistics			
Participant	*n*	Median posts per participant (IQR)	Median message length per participant (IQR)
Caregiver	141	18 (9–38)	11 (8–14)
Physician	10	245 (152–398)	12 (10–14)

Panel A reports message-level descriptive statistics within each consultation stage. Panel B reports participant-level summaries. Message length refers to the number of Chinese characters per message. Participant-level message counts and message lengths are summarized using the median and interquartile range (IQR).

Across the corpus, caregivers contributed 3,273 messages, compared with 2,665 messages from physicians, accounting for 55.1 and 44.9% of all coded messages, respectively. Caregiver and physician contributions varied across consultation stages (Table 4). During clinical data collection, caregivers produced 1,159 messages compared with 788 from physicians, whereas during diagnostic advice, caregivers produced 1,491 messages compared with 1,413 from physicians. Thus, both participant groups contributed most heavily during these two stages, although caregivers produced more messages overall.

Message length also varied by consultation stage and participant role. During problem initiation, caregiver messages were longer and more variable than physician messages, with median lengths of 9 and 3, respectively. The same pattern was observed during clinical data collection, although the difference was smaller (median = 10 for caregivers vs. 7 for physicians). Caregiver messages also showed greater dispersion in both stages, as reflected in their larger IQRs. These differences indicate that message-length distributions were not uniform across participant roles and consultation stages.

The pattern became more comparable during diagnostic advice. Both caregivers and physicians had a median message length of 10, while the mean lengths were 13.06 and 15.71, respectively. The corresponding IQRs were also similar (12 and 13). Thus, the contrast between longer caregiver messages and shorter physician messages was most evident during problem initiation and clinical data collection and was less pronounced during diagnostic advice.

During closure, message lengths decreased for both participant groups. Caregiver messages had a median length of 4, compared with 7 for physicians, while the corresponding IQRs were 5 and 6. Both groups contributed 311 messages during this stage. Taken together, these findings show that message volume and message length varied systematically across consultation stages. Caregivers contributed more messages overall and produced longer, more variable messages during the earlier stages, whereas message-length distributions became more comparable between caregivers and physicians during diagnostic advice. The results therefore indicate that participant differences in messaging were stage-dependent rather than uniform across the consultation.

### 4.3 Clarification sequences and the direction of informational work

The sequence analysis identified two forms of clarification, differentiated by their interactional direction and location in the consultation. As shown in Table 5, across the 347 consultation episodes, we identified 293 physician-led clarification sequences during Clinical Data Collection and 686 caregiver-initiated clarification sequences during Diagnostic Advice. These sequences contained 441 physician clarification message posts and 1,144 caregiver clarification message posts, respectively. Thus, a clarification sequence could contain more than one clarification-bearing message post, and the sequence counts and message-post counts are reported separately. During Clinical Data Collection, physician-led clarification involved follow-up questions through which physicians elicited additional information from caregivers. During Diagnostic Advice, caregiver-initiated clarification involved requests for further explanation, interpretation, or practical guidance following physicians’ assessments or recommendations.

**Table 5:** Quantitative summary of clarification sequences.

Clarification type	Consultation stage	Sequences	Clarification message posts
Physician-led	Clinical Data Collection	293	441(physician) 651(caregiver)
Caregiver-initiated	Diagnostic Advice	686	1,144(caregiver) 1,084(physician)
Total		979	

#### 4.3.1 Physician-led elicitation during clinical data collection

Across the 347 consultation episodes, 293 physician-led clarification sequences were identified during Clinical Data Collection, comprising 441 physician clarification message posts. These sequences involved physicians’ follow-up questions concerning symptoms, duration, frequency, associated symptoms, feeding practices, previous treatment, and other clinically relevant information. Rather than treating the initial caregiver report as a complete clinical account, physicians used follow-up questions to elicit additional information relevant to the ongoing assessment. The following extract illustrates one such sequence:

*Caregiver*: 宝宝这两天食欲不怎么好, 前天晚上把刚刚喝下去的奶全部吐了, 今天精神状态也不是特别好. *(The baby has not had much appetite these two days. The night before last, he/she vomited all the milk he/she had just drunk. Today his/her mental state is not particularly good either.)*

*Physician*: 昨天. 今天呕吐不?*(Yesterday? Is he vomiting today?)*

*Physician*: 拉肚子几次每天?*(How many times does he/she have diarrhea each day?)*

*Caregiver*: 今天没有呕吐, 但是每次喝完都会吐一点点, 吃得也比以前少. *(No vomiting today, but he spits up a little bit after each feeding, and eats less than before.)*

*Caregiver*: 粑粑是每天两次. *(He has bowel movements twice a day.)*

*Physician*: 喂养方面, 母亲饮食如何?*(Regarding feeding, how is the mother’s diet?)*

*Caregiver*: 混合喂养的, 我是上前天有一点拉肚子, 但是现在已经正常, 饮食也正常, 比较清淡. *(We do mixed feeding. I had a little diarrhea a couple of days ago, but now it’s back to normal, and my diet is normal too—relatively light.)*

*Physician*: 奶粉二次加热吗?*(Do you reheat the formula?)*

*Caregiver*: 没有, 喝不完就都是扔掉了的. *(No, if it’s not finished, we throw it away.)*

*Physician*: 嗯嗯, 可以益生菌调理一下. 热敷肚肚, 观察一下吧.

*(Okay, you can use probiotics to help regulate. Apply a warm compress to the tummy and observe for now.)*

The caregiver’s initial message presents several concerns, including reduced appetite, vomiting, and decreased energy, while information concerning the timing and recurrence of vomiting, bowel-movement frequency, feeding practices, maternal diet, and formula preparation is not yet provided. Rather than immediately providing a diagnostic assessment, the physician uses a series of targeted questions to obtain additional information. The questions address the timing and recurrence of vomiting, frequency of bowel movements, feeding practices, maternal diet, and formula-handling practices.

Importantly, these questions are sequentially connected rather than functioning as isolated requests for information. The caregiver’s responses provide new information that enables the physician to move to additional aspects of the child’s condition. For example, the initial report of vomiting is followed by clarification of whether vomiting has continued, while the report of reduced appetite is accompanied by information concerning bowel movements and feeding. The physician subsequently shifts to questions about feeding practices and formula preparation. Through this sequence, the initial broad description is progressively differentiated into information concerning symptom occurrence, frequency, feeding pattern, and relevant caregiving practices.

The interaction illustrates how clinically relevant information is progressively developed through caregiver responses to physician questions. Caregivers contribute experiential observations, while physicians use follow-up questions to elicit dimensions that are not explicitly provided in the initial account. The sequence moves from a relatively open-ended symptom description toward a more clinically structured account, after which the physician provides management advice.

This pattern suggests that physician-led clinical elicitation performs an information-structuring function during the clinical data-collection stage. Rather than merely generating additional messages, repeated questioning elicits information concerning specific clinical dimensions that are not contained in the initial report.

#### 4.3.2 Caregiver-initiated clarification following diagnostic and treatment advice

During Diagnostic Advice, we identified 686 caregiver-initiated clarification sequences, comprising 1,144 caregiver clarification message posts. These sequences involved caregivers’ requests for further explanation, interpretation, or practical guidance following physicians’ diagnostic assessments, treatment recommendations, or management advice. In contrast to physician-led clarification during Clinical Data Collection, these sequences involved caregivers seeking additional information after physicians had provided an assessment or recommendation.

The following consultation illustrates how this type of clarification unfolded interactionally. The caregiver initially reports fever, poor appetite, and the absence of bowel movements for two days:

*Caregiver*: 娃娃昨天早上38.3, 查了血正常. 昨天到今天反复低烧37.3左右. 胃口不好, 两天没有拉粑粑, 以前是每天早上起来第一件事就是拉粑粑. 今天晚上睡觉一直翻来覆去的, 一会又哭一会又哭. *(My baby had a temperature of 38.3 yesterday morning, and the blood test came back normal. From yesterday to today, there has been a recurring low-grade fever around 37.3. Poor appetite, and no bowel movement for two days. Previously, the first thing every morning was to have a bowel movement. Tonight, while sleeping, he/she keeps tossing and turning, crying on and off.)*

*Physician*: 有点便秘, 临时用一次开塞露, 吃药没有. *(A bit constipated. Use a glycerin enema once as a temporary measure. Have you given any medication?)*

*Caregiver*: 吃了胃蛋白酶和益生菌. *(pepsin and probiotics.)*

*Caregiver*: 开塞露咋个用?*(How do I use the glycerin enema?)*

*Physician*: 退热贴贴上. 其他药继续用. *(Put on a fever patch. Continue with the other medications.)*

*Caregiver*: 好的. 他这个必须要窝了粑粑才有好转是不是?*(Okay. So he can only get better after a bowel movement, right?)*

*Caregiver*: 晚上说了两次要窝粑粑, 结果坐马桶都没有拉出来. *(He said twice tonight that he needed to poop, but could not pass anything even sitting on the toilet.)*

*Physician*: 用一次开塞露会好点. *(Using one glycerin enema will help.)*

*Caregiver*: 好的, 益生菌和胃蛋白酶继续吃嘛?*(Okay, should we continue with the probiotics and pepsin?)*

*Physician*: 是的. *(Yes.)*

The physician’s response provides a relatively compressed clinical interpretation (“有点便秘” A bit constipated), together with a treatment recommendation to use a glycerin enema temporarily. The caregiver then immediately requests clarification concerning how the recommended treatment should be administered (“开塞露咋个用” How do I use the glycerin enema?). The subsequent exchange shows that the caregiver’s informational needs extend beyond the initial recommendation. The caregiver asks whether bowel movement is necessary for improvement, reports that the child has attempted to defecate without success, and later explicitly confirms whether the previously used medications should be continued.

The sequence therefore contains several successive caregiver-initiated clarification moves. The first concerns the practical implementation of treatment, while the subsequent questions concern the expected relationship between bowel movement and symptom improvement and the continuation of existing medication. These questions show that show that, in this episode, the caregiver sought additional procedural and interpretive information beyond the initial diagnostic and treatment advice.

At the same time, the physician’s responses remain relatively concise. The response to the question about the enema does not provide a detailed administration procedure; instead, the physician subsequently states that one use should help. Similarly, the question concerning continued use of probiotics and pepsin is answered simply with “是的Yes.” The exchange therefore illustrates a pattern in which a concise clinical recommendation is followed by caregiver attempts to obtain additional operational and interpretive detail, while the physician’s responses remain comparatively brief.

This does not necessarily indicate a failure of the consultation. The physician does respond to the caregiver’s questions, and the final exchange provides confirmation concerning continued medication. However, the sequence demonstrates that the informational requirements of the caregiver may extend beyond the initial diagnostic and treatment formulation. In an asynchronous messaging environment, a short recommendation can therefore generate additional clarification work when caregivers need to translate clinical advice into concrete decisions about what to do at home.

#### 4.3.3 Directional shift in informational work

Taken together, the 293 physician-led clarification sequences and 686 caregiver-initiated clarification sequences reveal a shift in the direction of clarification across consultation stages. During clinical data collection, caregivers provide descriptions of symptoms and circumstances, while physicians use follow-up questions to elicit and specify additional information. During diagnostic advice, physicians provide clinical assessments and recommendations, while caregivers may initiate further questions to interpret, clarify or operationalize those recommendations.

The two forms of clarification can therefore be understood as complementary interactional practices. In physician-led clarification, the interaction moves from caregivers’ experiential descriptions toward more specific clinical information. In caregiver-initiated clarification, the interaction moves from physicians’ clinical assessments or recommendations toward practical understanding and implementation.

Overall, the analysis suggests that clarification work changes direction across the consultation. Physicians primarily use questions to elicit and specify caregiver-provided information during Clinical Data Collection, whereas caregivers subsequently seek additional explanation or practical guidance following diagnostic and treatment advice. Thus, the asynchronous messaging environment does not eliminate clarification work; rather, it distributes clarification work differently across participants and consultation stages.

## 5 Discussion

### 5.1 Reconfiguring the architecture of institutional talk in asynchronous pediatric care

This study examined WeChat-based pediatric consultations as digitally mediated institutional interactions and identified a recurrent four-stage structure consisting of problem initiation, clinical data collection, diagnostic advice, and closure. From a digital public health perspective, this structure shows how access to pediatric healthcare through an asynchronous messaging platform is accompanied by a reorganization of the communicative activities through which health concerns are presented, clarified, assessed, and addressed. This finding extends established research on the structured organization of medical consultations by showing that the institutional functions of clinical encounters remain recognizable in an asynchronous messaging environment, while their sequential boundaries are reorganized. Classic research on institutional interaction has demonstrated that institutional encounters are organized around distinctive goals, participant roles, and interactional constraints (11). In medical settings specifically, consultation has traditionally been conceptualized as a structured sequence of activities through which participants establish the presenting problem, gather relevant information, formulate a clinical assessment, provide treatment, and terminate the encounter (12, 13). Rather than reproducing this conventional six-stage model as a series of clearly separated activities, the consultations in the present corpus frequently compressed opening with illness presentation and diagnosis with treatment recommendation. The resulting four-stage structure therefore reflects a reorganization of institutional activities rather than the disappearance of conventional consultation functions.

This finding is consistent with previous research showing that medical consultations constitute a distinctive institutional activity type characterized by recognizable goals, participant roles, and interactional expectations (11, 12). In conventional face-to-face encounters, opening, information gathering, diagnosis, treatment, and closure can often be identified through relatively recognizable sequential transitions (13, 30). Robinson (2003), in particular, demonstrated that acute medical visits are organized around an ordered project of establishing the reason for the visit, gathering additional clinical information, delivering a diagnosis, and providing treatment recommendations. The four-stage organization identified in the present corpus broadly corresponds to this functional logic, although some conventional activities are compressed or combined. Caregivers may initiate a consultation by simultaneously addressing the physician and describing the child’s symptoms, while physicians may combine clinical assessment with management recommendations within the same response. The functional logic of the consultation is therefore retained even when the boundaries between activities become less distinct.

Importantly, the present findings also resonate with more recent research questioning the assumption that clinical consultations consistently conform to idealized sequential models. A recent study (31), for example, found that although medical consultations broadly followed an expected chronological organization, fewer than one-third of the consultations they examined contained all six proposed phases, and many contained phases that were intertwined or occurred in an unexpected order. Such findings suggest that consultation models should be understood as functional and interactional frameworks rather than rigid scripts. The four-stage structure thus captures the recurrent functional organization of the consultations while allowing for local variation in how individual activities are realized.

The findings also contribute to research on computer-mediated and telehealth communication by demonstrating that technological mediation does not simply remove institutional structure. Instead, it can redistribute and compress the interactional work through which institutional activities are accomplished. Research on telemedicine has shown that mediated encounters can require participants to renegotiate interactional roles and communicative practices because the technological environment changes the conditions under which interaction takes place (32). More broadly, research on healthcare interaction has emphasized that the organization of medical activities is closely tied to how participants accomplish institutional tasks through interaction (33). In the present corpus, the asynchronous nature of messaging permits caregivers to send multiple contributions before receiving a physician response and allows physicians to address several preceding messages in a consolidated response. As a result, the organization of the consultation becomes distributed across multiple messages rather than being realized through a tightly coordinated sequence of contiguous turns.

For digital public health, this temporal reorganization is important because asynchronous communication changes how health information can be contributed, revisited, and responded to within a consultation. In face-to-face consultation, the sequential organization of talk is supported by immediate turn-taking, co-presence, and relatively stable participation frameworks. In WeChat consultations, by contrast, message persistence and delayed response make it possible for participants to contribute information at different points in time and for a single physician response to retrospectively address several earlier caregiver messages. Consequently, the boundaries between consultation stages become more permeable. A caregiver’s initial message may simultaneously perform the functions of opening and problem presentation, while a physician’s response may simultaneously perform assessment, diagnosis, treatment recommendation, and partial closure. The resulting organization can therefore be understood as a mediated restructuring of institutional sequence, in which the functional architecture of medical consultation persists but the temporal and interactional realization of that architecture changes.

The four-stage structure identified here should therefore not be interpreted as a universal replacement for conventional consultation models. Rather, it represents the recurrent organization observed in this particular corpus of WeChat-based pediatric consultations. This interpretation is consistent with research showing that actual clinical encounters may diverge from idealized consultation structures and that phases may be omitted, combined, or intertwined in practice (30, 33). The findings further suggest that asynchronous messaging constitutes an important interactional condition under which such restructuring occurs. Institutional goals remain relatively stable—namely, identifying the child’s health problem, gathering clinically relevant information, providing an assessment and recommendation, and bringing the consultation to a close—while the interactional resources used to accomplish these goals are reshaped by the affordances of the messaging platform.

### 5.2 Redistributing observable informational work across caregiver and physician roles

The second major finding concerns the distribution of observable informational activities between caregivers and physicians. Caregivers contributed more messages overall than physicians, and their messages were particularly longer and more variable during problem initiation and clinical data collection. Physicians, in turn, contributed targeted clarification questions during clinical data collection and clinical assessments and management-oriented recommendations during diagnostic advice. These findings suggest that the central asymmetry in digital pediatric consultations is not simply an asymmetry in message quantity, but a differentiation in the observable communicative activities performed by participants across consultation stages.

This finding extends established research on medical communication showing that patients possess important experiential knowledge of their symptoms and illness experiences, while physicians occupy institutionally privileged positions with respect to diagnosis, treatment, and clinical decision-making (34, 35). Research on epistemics in medical interaction has similarly demonstrated that patients and physicians may possess different epistemic rights: patients have primary access to their own illness experiences, whereas physicians claim greater epistemic authority over diagnosis and treatment (36). In the present corpus, caregivers were the primary sources of information about children’s everyday health experiences, including symptoms, duration, severity, behavior, appetite, and responses to previous treatment. This information was frequently distributed across multiple messages rather than presented in a single contribution. Physicians subsequently used targeted questions to elicit particular dimensions of the caregivers’ reports and responded with clinical assessments and management-oriented recommendations. This pattern is consistent with recent work emphasizing that medical authority is not simply a matter of possessing more knowledge, but is interactionally accomplished through the evaluation, interpretation, and accountable use of information (37, 38).

The distinction between information provision and observable information elicitation and response is particularly relevant in pediatric consultations. Unlike adult patients who can directly report their own symptoms and experiences, caregivers frequently act as observers and proxies for children.

### 5.3 Clarification and the construction of clinically structured information

A particularly important contribution of the study is the identification of clarification sequences as a mechanism through which information relevant to clinical assessment is progressively specified through interaction. Caregivers’ initial reports frequently established the child’s presenting problem but did not immediately specify all dimensions relevant to clinical assessment. This pattern is consistent with research on medical interaction showing that patients’ presenting concerns are progressively organized through physicians’ opening questions (39, 40).

This finding addresses a gap identified in previous research on digital health communication. Existing studies of messaging-based healthcare have documented benefits such as accessibility, continuity, patient support, and patient-generated information sharing, but comparatively less attention has been given to the sequential processes through which information is elicited and specified for clinical purposes (41, 42). The present findings demonstrate that clarification is not merely a peripheral conversational activity. It is a recurrent interactional practice through which participants specify health-related information within an asynchronous consultation.

The process can be understood as a movement from initial experiential reporting toward progressively specified clinical information (12, 43). Caregivers typically report what they observe or experience—for example, that a child has a fever, cough, poor appetite, or changes in behavior. Physicians then introduce questions that organize these observations according to clinically relevant dimensions such as onset, duration, severity, associated symptoms, and functional status. The resulting information is not simply a more detailed version of the original account. It is information that has been reorganized through interaction for a particular clinical purpose (44).

This finding also helps explain the quantitative patterns observed in the study. During clinical data collection, caregivers produced more messages than physicians (1,159 vs. 780), and caregiver messages were longer and more variable in length. The sequence analysis provides an interactional context for this distribution: physicians’ clarification questions were followed by successive caregiver contributions that supplied additional information. Thus, the greater number of caregiver messages serve as a descriptive feature of how information was exchanged during this consultation stage.

From a digital health perspective, this finding identifies structured symptom prompts as a potentially useful area for further development. If physicians repeatedly elicit information concerning duration, severity, associated symptoms, or previous treatment, digital interfaces could potentially provide optional prompts that encourage caregivers to organize these dimensions before or during the consultation. Such prompts may help make relevant information more visible and reduce some forms of repeated elicitation. However, the present observational study does not establish that structured prompts would improve information completeness, reduce consultation time, or enhance clinical outcomes (42). Their effectiveness should therefore be treated as an empirical question for future intervention studies.

Importantly, the findings do not imply that caregiver reports are inherently deficient or that caregivers lack adequate knowledge about the child’s condition. Rather, they suggest that caregivers and physicians contribute different types of knowledge and perform different observable communicative activities (43). Caregivers contribute experiential and contextual knowledge, while physicians organize that knowledge according to clinical assessment requirements. Digital consultation systems may therefore benefit from supporting the transition between these forms of information without assuming that one form is inherently more valuable than the other.

### 5.4 Brief advice and digital health guidance

The analysis also identified a contrasting pattern during diagnostic advice. Physicians frequently combined clinical assessment and management recommendations into relatively concise responses, and some advice sequences remained brief even when caregivers explicitly requested further explanation. This pattern suggests a shift in the communicative activities performed across consultation stages: clinical data collection involved successive exchanges through which additional information was elicited and provided, whereas diagnostic advice was more often realized through assessment, recommendation, and subsequent clarification when caregivers sought further specification.

The message-length patterns are best understood in relation to these communicative functions. During diagnostic advice, caregivers and physicians had the same median message length of 10 words, with comparable variability (IQR = 12 and 13, respectively). This similarity indicates that message length alone does not distinguish the communicative roles of the two participants at this stage. Rather, their messages performed different functions: physicians primarily provided clinical assessments and management-oriented recommendations, whereas caregivers continued to provide information, ask questions, or seek clarification.

At the same time, some diagnostic advice sequences were followed by caregiver requests for additional explanation. When caregivers asked about the reasons for an assessment, the necessity of medication, expected symptom progression, or recommended next steps, the interaction continued through further clarification. This pattern highlights the importance of explanation and guidance alongside the provision of clinical recommendations. In asynchronous pediatric consultation, a recommendation may address an immediate management question while leaving additional aspects of the caregiver’s concern to subsequent interaction. This issue is particularly relevant to asynchronous pediatric care because the temporal organization of messaging changes how clarification can occur. In face-to-face consultation, caregivers can usually seek clarification immediately within the same encounter. In a messaging environment, additional clarification requires a further message and a subsequent response. The availability and clarity of guidance may therefore influence whether caregivers can readily identify what action to take, what symptoms to monitor, and when further medical assessment may be needed.

### 5.5 Implications for digital pediatric health service design

The interactional patterns identified in this study suggest several directions for the design of digital pediatric health services. Rather than treating technological features as independent of communication, the findings indicate that platform design can support particular forms of health-information exchange between caregivers and physicians.

First, structured symptom prompts may support the reporting of information that is frequently elicited during clinical data collection. The recurrent clarification sequences identified in the corpus show that physicians frequently asked about dimensions such as symptom onset, duration, frequency, associated symptoms, previous treatment, feeding, and current condition. Optional prompts covering these dimensions could provide caregivers with a preliminary framework for reporting health concerns before or during consultation. Such prompts may be particularly useful in pediatric care, where caregivers serve as the primary reporters of children’s symptoms and everyday health experiences.

Second, message threading and question-response linkage could support the traceability of information distributed across multiple messages. In the corpus, caregivers sometimes contributed different aspects of a child’s condition through successive messages, while physicians could address several preceding contributions within a single response. Interface features that visually connect questions with corresponding responses, or group messages according to symptoms or consultation topics, could make these relationships easier to follow. This may be particularly useful when several symptoms, medications, or concerns are discussed within the same consultation.

Third, explicit escalation guidance may strengthen the safety and usability asynchronous pediatric consultation. Some physician advice in the corpus included conditions under which caregivers should seek further medical evaluation. Such guidance can clarify the boundary between information and management that can be provided remotely and circumstances that require additional clinical assessment. Digital systems could support this function through clinician-customizable fields for warning signs, follow-up recommendations, and escalation criteria.

These design directions also point toward a broader principle for digital pediatric health services: communication architecture is part of the infrastructure through which health information becomes usable. Providing access to physicians through messaging platforms does not by itself determine how clearly questions, responses, recommendations, and follow-up needs are connected. The organization and visibility of messages can shape how caregivers locate relevant information and how physicians respond to information provided at different points in the consultation.

For digital public health, this perspective extends the discussion of access beyond the availability of digital consultation itself. An asynchronous service may increase opportunities for caregivers to obtain professional advice, but the usefulness of that access also depends on how effectively health information can be exchanged and acted upon. Designing pediatric digital health services therefore requires attention to both access and communicative usability. Future intervention studies could compare alternative combinations of symptom prompts, message-threading functions, and structured guidance to determine their effects on information exchange, clarification patterns, caregiver understanding, consultation efficiency, and subsequent care-seeking.

### 5.6 Scope, limitations, and future research

Several limitations define the scope of the present findings. First, the study is based on a cross-sectional corpus collected within a single year and focuses on complete consultation episodes. The analysis therefore provides an episode-level account of interactional organization rather than a longitudinal account of how communication patterns develop across repeated consultations. Future longitudinal research could examine whether clarification practices and message-production patterns vary across repeated interactions with the same service or physician and whether such patterns are associated with continuity of care.

Second, the study focuses on the organization of communication rather than the clinical validity of diagnoses or treatment recommendations. Although the corpus provides evidence concerning how physicians elicited information and communicated clinical assessments and recommendations, diagnostic accuracy was not independently evaluated. Future research could combine interactional analysis with clinical outcome measures to examine whether particular communication patterns are associated with diagnostic agreement, appropriate follow-up, or other indicators of care quality.

Third, caregiver trust, satisfaction, understanding, and perceived quality of care were not directly measured in the present corpus. The occurrence of caregiver-initiated clarification following diagnostic advice provides evidence that further specification was sometimes sought, but the interaction itself does not reveal the caregiver’s underlying evaluation of the preceding response. Future mixed-methods research combining conversation analysis with caregiver interviews, surveys, or validated measures could investigate how clarification experiences relate to perceived understanding, trust, satisfaction, and confidence in subsequent care decisions.

Fourth, the study does not provide direct evidence concerning clinician workload, service efficiency, operational costs, or long-term platform use. These dimensions are important for assessing the implementation and sustainability of digital pediatric health services but require organizational, economic, and longitudinal data. Future research could therefore combine interactional measures with consultation duration, clinician workload, service utilization, and cost-related indicators.

Fifth, the corpus was drawn from a hospital-associated WeChat consultation group in China. The interactional patterns identified here are therefore situated within a particular institutional, technological, and cultural context. The organization of asynchronous pediatric consultation may differ across platforms, healthcare systems, regions, and populations. Comparative research involving different digital platforms and healthcare settings would help determine which aspects of the observed interactional organization are specific to this context and which may be more broadly characteristic of asynchronous pediatric communication.

Future research could build on these findings through three complementary directions. First, experimental studies could examine whether structured symptom prompts affect the distribution of caregiver-provided information and the frequency or type of clarification sequences. Second, interface research could investigate whether message threading, topic grouping, or structured advice formats improve the traceability and usability of health information. Third, mixed-methods and longitudinal studies could connect interactional patterns with caregiver understanding, trust, satisfaction, care-seeking decisions, and continuity of care.

These directions would also allow future studies to examine the public health implications of asynchronous pediatric consultation more directly. In particular, research could investigate whether improvements in digital health-information exchange contribute to more appropriate care-seeking, better caregiver health-information understanding, improved continuity of care, or more equitable access to pediatric advice. Such outcome-oriented research would complement the interactional evidence provided by the present study and help clarify how communication practices within digital consultations relate to broader health-service outcomes.

## 6 Conclusion

This study examined how pediatric medical consultations are organized within an asynchronous WeChat-based group messaging environment, using a corpus of 347 complete consultation episodes containing 5,938 analyzable messages. The analysis identified a recurrent four-stage interactional structure—problem initiation, clinical data collection, diagnostic advice, and closure—that preserves the functional logic of conventional medical consultations while demonstrating how asynchronous messaging compresses or redistributes traditionally distinguishable activities. Opening and illness presentation were frequently combined into problem initiation (241/347 episodes, 69.45%), while diagnosis and treatment recommendation were commonly co-realized as diagnostic advice (298/347 episodes, 85.88%). All 347 complete episodes contained observable instances of all four adapted stages.

The findings further demonstrate that caregiver and physician contributions differ systematically across consultation stages. Caregivers produced more messages overall (3,273 vs. 2,665) and contributed longer, more variable messages during problem initiation and clinical data collection, whereas physicians’ messages became more substantial during diagnostic advice. Clarification sequences—293 physician-led sequences during clinical data collection and 686 caregiver-initiated sequences during diagnostic advice—show how participants progressively specified clinically relevant information through successive exchanges. These patterns reveal a redistribution of observable communicative activities across participant roles and consultation stages rather than a simple asymmetry in message quantity.

From a digital public health perspective, these findings have implications for the design of asynchronous pediatric consultation services. Structured symptom prompts could support caregivers in reporting information frequently elicited during clinical data collection. Message threading and question-response linkage could improve the traceability of information distributed across multiple messages. Explicit escalation guidance could clarify the boundary between remote management and circumstances requiring in-person assessment. These design directions should be tested through future intervention studies rather than treated as established solutions on the basis of the present observational evidence. Several limitations should be considered when interpreting these findings. The cross-sectional corpus provides episode-level interactional evidence but does not establish diagnostic accuracy, caregiver trust, satisfaction, understanding, retention, or long-term service sustainability.

The findings are based on 347 complete episodes that exhibited clear closure markers, and therefore pertain only to consultations with an identifiable interactional completion. Future research could combine interactional analysis with experimental designs, longitudinal data, and mixed methods to examine whether communication design features influence information completeness, caregiver understanding, consultation efficiency, care-seeking decisions, and continuity of care. Comparative research across platforms, healthcare systems, and populations would also help determine which aspects of the observed interactional organization are context-specific and which may be more broadly characteristic of asynchronous pediatric communication.

Overall, this study demonstrates that asynchronous digital pediatric consultation is not simply a conventional medical encounter transferred to a messaging platform. Rather, the messaging environment reorganizes how institutional activities are sequenced and how communicative work is distributed between caregivers and physicians. The central challenge is not merely how much information is exchanged, but how health-related information is progressively elicited, clarified, specified, and translated into actionable guidance within the temporal and sequential constraints of asynchronous messaging. By identifying these interactional processes, the study provides an empirical basis for further research on how digital communication environments can support more coherent, understandable, and appropriately bounded pediatric healthcare interactions.

## Data Availability

The datasets used and/or analyzed during the current study are available from the corresponding author on reasonable request. Requests to access these datasets should be directed to sunxue@swmu.edu.cn.
